# Developing the Autism Model of Implementation for Autism spectrum disorder community providers: study protocol

**DOI:** 10.1186/1748-5908-7-85

**Published:** 2012-09-10

**Authors:** Amy Drahota, Gregory A Aarons, Aubyn C Stahmer

**Affiliations:** 1 Department of Psychology, San Diego State University, San Diego, CA, USA; 2 Child and Adolescent Services Research Center, Rady Children’s Hospital, San Diego, CA, USA; 3 Department of Psychiatry, University of California, San Diego, La Jolla, CA, USA

**Keywords:** Autism spectrum disorder, Evidence-based practice, Implementation, Organization, Community provider, Model development

## Abstract

**Background:**

Currently, 1 out of 88 children are diagnosed with an autism spectrum disorder (ASD), and the estimated cost for treatment services is $126 billion annually. Typically, ASD community providers (ASD-CPs) provide services to children with any severity of ASD symptoms using a combination of various treatment paradigms, some with an evidence-base and some without. When evidence-based practices (EBPs) are successfully implemented by ASD-CPs, they can result in positive outcomes. Despite this promise, EBPs are often implemented unsuccessfully and other treatments used by ASD-CPs lack supportive evidence, especially for school-age children with ASD. While it is not well understood why ASD-CPs are not implementing EBPs, organizational and individual characteristics likely play a role. As a response to this need and to improve the lives of children with ASD and their families, this study aims to develop and test the feasibility and acceptability of the Autism Model of Implementation (AMI) to support the implementation of EBPs by ASD-CPs.

**Methods/design:**

An academic-community collaboration developed to partner with ASD-CPs will facilitate the development of the AMI, a process specifically for use by ASD community-based agencies. Using a mixed methods approach, the project will assess agency and individual factors likely to facilitate or hinder implementing EBPs in this context; develop the AMI to address identified barriers and facilitators; and pilot test the AMI to examine its feasibility and acceptability using a specific EBP to treat anxiety disorders in school-age children with ASD.

**Discussion:**

The AMI will represent a data-informed approach to facilitate implementation of EBPs by ASD-CPs by providing an implementation model specifically developed for this context. This study is designed to address the real-world implications of EBP implementation in ASD community-based agencies. In doing so, the AMI will help to provide children with ASD the best and most effective services in their own community. Moreover, the proposed study will positively impact the field of implementation science by providing an empirically supported and tested model of implementation to facilitate the identification, adoption, and use of EBPs.

## Background

Autism spectrum disorders (ASD) are a set of pervasive, clinically complex disorders that require multiple intervention types to alleviate common clinical targets, such as impaired social skills and communication, executive functioning, empathy and perspective taking, sensory perception, and motor skills, along with restricted and circumscribed interests and co-occurring psychiatric disorders [1]. Currently 1 out of 88 children are diagnosed with an ASD [2], and treatments for ASD symptoms are estimated to cost $125 billion annually [3]. Typically, ASD community providers (ASD-CPs) provide services to children with any severity of ASD symptoms using a combination of various treatment paradigms, with some treatments considered efficacious or probably efficacious and some with no evidence-base [4-7]. When evidence-based practices (EBPs) are successfully implemented by ASD-CPs, outcomes include increased IQ, communication, social and daily living skills, and provide an improved developmental trajectory for children with ASD [8]. Despite this promise, EBPs are often implemented unsuccessfully, and other treatments used by ASD-CPs lack supportive evidence; both may be inconsequential at best and harmful, even deadly, to children with ASD, at worst [9,10]. Given that 25.3% of California Department of Developmental Services’ total budget pays for services for individuals with ASD [11], it is critical that EBPs be successfully implemented by ASD-CPs to improve the lives of children with ASD and their families, and as a measure of fiscal responsibility.

Recently, research emphasis has been placed on developing and testing EBPs for children with ASD and their families, resulting in an increase in the number of EBPs targeting many common clinical targets of the disorder [2,12]. However, ASD-CPs are not implementing these EBPs at the same rate that they are being developed and tested [5]. Instead, ASD-CPs are either delivering a single type of intervention for all clinical targets (*e.g.*, Discrete Trial Teaching (DTT)), combining practices in a non-systematic manner, or using practices that lack supportive evidence. For example, DTT is a highly structured EBP that targets ASD symptoms, such as communication, and has robust evidence supporting its use. However, it does not fit all children with ASD, in particular, higher functioning school-age children with ASD, nor treat many common clinical disorders experienced by children with ASD [6]. Thus, many children with ASD in the community are left with unmet clinical needs [7].

It is not well understood why ASD-CPs are not implementing EBPs for common clinical issues related to ASD. It is likely that both organizational and individual ASD-CP characteristics play a role in hindering implementation. Further, preliminary individual interviews with a convenience sample of five ASD community agency leaders (herein referred to as ‘ASD leaders’) indicated a need for a contextually specific model designed for use by ASD-CPs to facilitate implementing EBPs for children with ASD. That is, while EBPs may be available, ASD-CPs do not have an efficient and effective process to facilitate EBP adoption, implementation, and sustainment. As a response to this need, and to improve the lives of children with ASD and their families, this study aims to develop the Autism Model of Implementation (AMI) that will facilitate implementation of EBPs within ASD community-based agencies.

### A contextually specific implementation model for ASD-CPs is necessary

Current empirically derived implementation models [13-15] provide useful guidance for the development of the AMI given that they consider a comprehensive set of organizational and individual provider factors likely to contribute to the adoption and implementation of EBPs. However, it is not known whether these models can be generalized to ASD-CPs. Attempting to generalize empirically derived implementation models for other settings may fail to recognize the unique context and characteristics of ASD-CPs. For example, compared with other community-based contexts (*e.g.*, community mental health providers), ASD-CPs differ in training, attitudes towards EBPs, and funding [5,16,17]. Preliminary data gathered for this study indicate that ASD Leaders are willing to adopt EBPs (after evaluating the EBP-agency fit) but identified implementation barriers ranging from the educational level of their staff (predominantly undergraduate college students), funding (*e.g.*, need for ongoing intervention to treat pervasive symptoms, insurance limits treatment sessions and types of treatments, so many families must pay out-of-pocket for many services), societal constraints (*e.g.*, parents may demand specific services that are not typically provided for specific symptoms of ASD), and difficulty identifying EBPs for use with children with ASD. ASD-CPs may be expected to adapt interventions to meet the needs of the heterogeneous set of symptoms and behaviors displayed by children with ASD without appropriate training or knowledge of the intervention. Further, parents and consumer advocacy groups have a strong influence on legislation and funding for services, thus affecting the organizational context of ASD agencies [18].

### Collaboration with community partners

Traditional efforts to implement EBPs in community settings have followed a research-to-practice approach to implementation, wherein researchers disseminate new EBPs to ASD-CPs in a unidirectional manner from the laboratory to community and expect practitioners to adopt, implement, and adhere to EBP protocols without it being tailored for the agency context or ASD-CPs characteristics, or inviting ASD-CPs to participate in the development of, and decision-making about the protocol [19]. Consequentially, efforts often result in failed implementation, with providers reporting a lack of investment in the EBP and focusing on different needs than those being addressed by the EBP [20,21]. These challenges highlight the need for improved collaboration between academia and community stakeholders [22]. Numerous collaborative partnership models exist, including community-based participatory research (CBPR) [21], community-participatory partnered research (CPPR) [23], participatory action research (PAR) [24], and academic-community collaboration (ACC) [19,25]. Because this project is researcher-initiated, the ACC model will be used. The ACC model is characterized by: shared vision and impact benchmarks, building interdependence between collaborative partners, consensus and shared decision making, and formalized collaborative structure (*e.g.*, roles, responsibilities of collaborators). ACC is an ideal approach to overcome previous implementation challenges because it involves community stakeholders as partners in most aspects of research endeavors [19,25] and will allow researchers to better address issues of external validity, feasibility, and acceptability of the AMI.

### The autism model of implementation (AMI)

While numerous empirically-derived models exist that are comprised of variables affecting adoption and implementation of EBPs [13-15], no models focus on implementation of EBPs by ASD-CPs, specifically. Further, few studies have conducted applied research to assess the outcomes of these models of implementation in practice. In the few studies investigating the application of implementation models [26,27], key elements leading to success have emerged, including: using distinct phases to guide implementation; involving direct service providers and community members as collaborative partners providing input on the uptake of EBPs; and providing comprehensive training with ongoing support prior to and during early EBP uptake.

**Figure 1:**
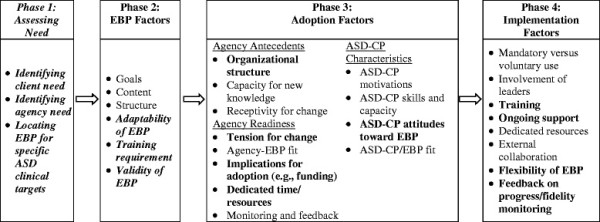
**Preliminary conceptual model of the Autism Model of Implementation (Adapted from Aarons*et al*., 2011**[13]**; Damschroeder*et al*., 2009**[14]**; and Greenhalgh*et al*., 2004**[15]**).** Note. Discussions with ACC Partners yielded: factors to be emphasized (bolded) and added (bolded and italicized).

The purpose of this study is to develop the AMI that will involve factors relevant to implementation on multiple levels, including assessing both agency factors and provider characteristics related to facilitating or inhibiting implementation of EBPs (*e.g.*, structured needs assessment; standardized process for evaluating the EBP, evaluating organizational adoption readiness factors). In collaboration with ACC partners, a review of the literature and agency implementation assessment data will guide the development of the AMI. Preliminary discussions with ASD-CPs indicate that some factors included in general health service organizations’ models of implementation may be of particular importance to the implementation of EBPs by ASD-CPs (*e.g.*, ongoing support, flexibility of EBP) and require greater emphasis in the AMI. Furthermore, additional factors may need to be added to the AMI (*e.g.*, assess need factors). Although the exact factors included in the AMI will require completion of aim one research activities, Figure 1 presents a preliminary model that has been adapted in collaboration with ACC partners. In particular, questions remain about a feasible model and process of implementation, such as: within an ASD community-based agency, who assesses client and agency need and how; who identifies the appropriate EBP and how; and what structures and processes can be embedded within the AMI regarding evaluating the validity, feasibility, or adaptability of the EBP? What agency-level training process will facilitate EBP implementation? What structured evaluation of implementation is needed to sustain the use of the EBP by ASD-CPs over time? Results from aim one will be discussed with ACC partners in order to refine the preliminary AMI model.

The work in the proposed project will proceed in three phases corresponding to three specific aims:

• Aim one: Assess agency antecedents and readiness factors, individual ASD-CP characteristics, and methods of implementing EBPs by ASD Leaders to identify factors that facilitate or hinder adoption and use of EBPs by ASD-CPs.

• Aim two: Develop the autism model of implementation (AMI) with the focus on identification, adoption, and use of EBPs within community-based agencies serving children with ASD.

• Aim three: Examine the feasibility and acceptability of the AMI through a small-scale pilot study. It is hypothesized that ASD-CPs will successfully complete each activity within each phase of the AMI to implement new EBPs within their ASD community-based agencies.

### Methods/Design

Aim one: Assess agency antecedents and readiness factors, individual ASD-CP characteristics, and methods of implementing EBPs by ASD Leaders to identify factors that facilitate or hinder adoption and use of EBPs by ASD-CPs.

### Participants

At least two focus groups involving approximately five to eight ASD-CPs will be conducted to assess perspectives related to EBP implementation factors. ASD-CPs will include ASD agency Leaders as well as direct service providers, and are expected to be mostly female and range in educational level from college students to Ph.D. level staff. Age is not known, but typically varies between 24 and 69 years [5]. It is expected that ethnical/racial composition will reflect that of mental health service providers in San Diego, Imperial, and Orange Counties, which is diverse.

### Procedure

Prior to the focus group, the principal investigator (PI) will conduct a comprehensive literature review and identify theoretically derived implementation factors that are likely to be relevant for the AMI [13-15]. Once identified, implementation factors will be further examined for applicability by using a mixed method research design involving quantitative measures (see below) and focus groups of ASD-CPs. Findings will be used to expand or narrow the focus of each theoretically derived implementation factor. Specifically, focus group participants will be asked to complete and return the quantitative measures prior to the focus group in order to allow focus group discussions to elaborate on the responses. Focus groups will be convened at times convenient to a majority of participants to elicit perceptions of identified implementation factors as well as previously unidentified factors particular to ASD-CP services. Further, focus groups will evaluate what methods are being used, if any, to assess need, evaluate EBPs, make decisions regarding adoption, and facilitating implementation of EBPs.

## Measures

### Demographic survey

The demographic survey will provide information about the ASD-CPs individual characteristics including gender, race/ethnicity, educational level, years as an ASD-CP at and outside of their current ASD agency, training received at the ASD agency, training received outside of the ASD agency, and professional or networking organization membership.

### Evidence-based practice attitude scale (EBPAS)

Focus group participants will complete the EBPAS [28,29], a structured questionnaire designed to assess empirically-derived constructs related to adoption and implementation of EBPs: appeal, requirements, openness, divergence, and a total EBPAS score representing the ASD-CPs global attitude toward EBPs [30,31].

### Children’s services survey (CSS)

The CSS [32], adapted for use by ASD-CPs, will assess organizational culture and climate in order to provide information about factors related to implementing EBPs.

### ASD community provider strategies survey (PSS)

The PSS, adapted for this study, will assess ASD-CPs’ use of EBPs, knowledge of the evidence supporting the use of the practice, and whether they use the EBP or not, and whether the have received formal training in the EBP [33].

### Focus group data

Semi-structured focus group guides will be used with ASD-CPs to elicit data on their perspectives on the following topics: needs of both clients and ASD-CPs in regard to children with anxiety disorders; EBPs in general; agency and ASD-CP factors that might facilitate or inhibit EBP implementation; and their current process used to implement EBPs (if any). Focus groups will be conducted until the data collected reaches saturation (*i.e.*, the same information is obtained from more than one group). The PI will introduce the initial issues to be addressed and moderate the dynamics of the group discussion to assure that all views are represented. This structure is dictated by the objective of collecting comparable data from both focus groups. Focus group discussions will follow a ‘funnel interview structure’ [34], starting with broader researcher-driven issues and narrowing to more participant-driven specific illustrations of these issues.

### Analysis plan: Integration of qualitative and quantitative analyses

Integrating qualitative and quantitative methods involves both deductive and inductive approaches to the research design and interpretation of data within the same study [35], and is most appropriate for addressing the complexities of modern social phenomena by utilizing multiple approaches to understand a given phenomenon or process [36]. This study will employ a mixed method approach in order to acquire understanding of the factors involved in implementation by ASD-CPs, and to evaluate the process of implementation that ASD-CPs are currently using in order to identify facilitating and inhibiting factors related to implementing EBPs by ASD-CPs. Implementation factors are not easily quantified, requiring the use of qualitative methods and data to inform the development of structured assessment and evaluation tools (quantitative measures) for future use. Three different strategies will be employed when integrating quantitative and qualitative data, as suggested by Creswell and Plano Clark [37]: triangulation, expansion, and complementarity.

First, merging qualitative and quantitative data will occur through triangulation in which results of quantitative and qualitative analyses are placed side by side to determine whether each provides the same answer to the same question (convergence; *e.g.*, do ASD-CP focus group data concur with quantitative data regarding attitudes regarding EBPs, organizational culture and climate, and EBP use and knowledge). Second, results from qualitative and quantitative analyses will be linked when the former is used to provide explanations for unanticipated findings produced by the latter (expansion; *e.g.*, questionnaire data will be used to assess the prevalence of emergent barriers to implementing EBPs observed in qualitative interviews). Finally, results of qualitative analysis can be embedded within the analysis of quantitative data by helping to contextualize results obtained in statistical analyses focused on the questionnaires (complementarity).

Aim two: Develop the autism model of implementation (AMI) with focus on identification, adoption, and use of EBPs within agencies serving children with ASD.

### Participants

Development of the AMI will involve approximately seven ACC partners. The ACC will be comprised of three to five ASD-CPs who are responsible for directing, supervising, or training direct service providers within their agencies, one to two funding agency representatives (*e.g.*, regional centers, insurance companies, school districts) who are knowledgeable about how funding decisions are made for children with ASD, and an implementation researcher with expertise in studying implementation factors related to community agencies. A systematic recruitment strategy will be used in order to maximize the diversity of ACC partners, which is expected to increase the generalizability of the AMI to a variety of organizations, geographic locations, and providers.

### Procedure

In collaboration with ACC partners, the review of the literature and aim one data will guide the development of the AMI. Specifically, results will be presented to ACC partners to obtain feedback and additional interpretation. Results and ACC partner feedback will be used to guide the development of the AMI and AMI materials (*e.g.*, needs assessment materials, *et al*.). Although the exact factors included in the AMI will require completion of aim one research activities, the AMI will likely include four phases: assessing needs of children with ASD and the agency, examining EBP factors to address those needs, addressing organizational factors and ASD-CP characteristics related to adoption, and identifying implementation factors, such as training, EBP flexibility, and ongoing support that will facilitate EBP use at the agency (Figure 1). For each phase, the AMI will have specific products, developed by the PI in collaboration with ACC partners, to be used by ASD Leaders in order to accomplish the goals of that phase. For example, ACC partners who collaborated in the development of this proposal reported that assessing need was an important step for an implementation model and that there was not a generalized, systematic assessment tool available for use by ASD-CPs. Therefore, an organizational assessment tool to identify common clinical targets of school-age children with ASD will be developed to support the implementation of the AMI. Table 1 outlines likely materials supporting AMI implementation.

**Table 1:** AMI Process Materials for the AMI preliminary conceptual model

**AMI Phase**	**AMI Process Materials**
Phase 1: Assessing Need	· Assessment to identify common clinical targets of school-age children with ASD.
	· Assessment to identify agency need.
	· Guidance for locating EBPs to meet common clinical targets of school-aged children with ASD through targeted search engines cataloguing EBPs, such as National Standards Project, NREPP, Promising Practices Network, and PracticeWise.
Phase 2: EBP Factors	· Structured evaluation process to identify the goals, content, and structure of the EBP, rate the adaptability of the EBP, and training requirements.
	· Structured guide for evaluating the validity/evidence supporting the use of the EBP.
Phase 3: Adoption Factors (including preparing for uptake)	· Recommendations for enhancing agency antecedents and readiness for adoption of EBPs.
	· Assessing fit between goals of the EBP and agency values/mission.
	· Structured tool for assessing the feasibility of the EBP for use by ASD-CPs.
	· Process for adapting the EBP for use by ASD-CPs without reducing effectiveness, as needed.
	· Decision tree involving evaluation of the implications of adoption including personnel, dedicated time/resources, initial training, ongoing support, materials cost, etc. (e.g., cost-benefit calculator).
	· Structured staff activity related to assessing ASD-CP motivation, existing skills and capacity, attitude toward EBP, and fit.
Phase 4: Implementation Factors	· Planning tools for update of EBPs including training, ongoing support, adapting EBP, and fidelity monitoring of EBP use.
	· Re-evaluation of needs assessment.

### Process of developing the AMI

**Figure 2:**
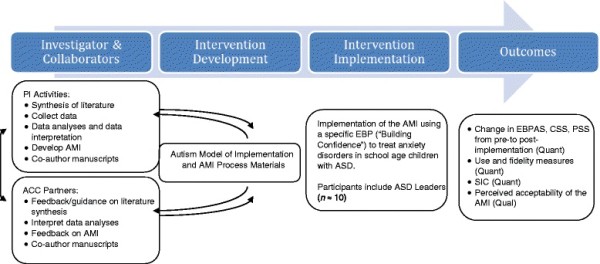
Process model of AMI development and pilot test.

The development of the AMI will involve an iterative approach (Figure 2) involving the ACC partners. Specifically, the PI will revise the preliminary conceptual model of the AMI based on the literature review and aim one results. The ACC partners will provide feedback to enhance the interpretation of aim one results and for revisions to the AMI and AMI process materials. It is expected that this iterative approach will increase the external validity, feasibility, and acceptability of the AMI.

Aim three: Examine the feasibility and acceptability of the AMI through a small-scale pilot study.

The AMI model will be designed for use by ASD community-based agencies to implement any EBP. However, for this pilot study, an EBP has been chosen *a priori*. This will allow for examination of each phase of the AMI and preliminary comparison between use of the AMI among agencies who participate in the pilot study. Specifically, an EBP to treat co-occurring anxiety disorders in children with ASD has been targeted due to the prevalence of anxiety disorders among school-age children with ASD and interest expressed by ACC partners.

Clinically significant anxiety disorders affect up to 80% of school-age children with ASD, causing heightened impairment above and beyond that caused by the ASD, and is the second most highly prevalent problem reported by parents [38-43]. Cognitive behavioral therapy (CBT) to treat anxiety disorders in children with ASD is considered evidence-based because multiple laboratories using rigorous research designs and methods (*e.g.*, randomized controlled trials) have found evidence supporting its use to alleviate or reduce anxiety symptoms or interference with large treatment effect sizes at post-treatment compared to control conditions [44-46]. Moreover, evidence suggests that intervening on children’s anxiety disorders has distal outcomes such as increasing social and daily living skills, and family functioning [44,47,48]. Preliminary data gathered from ASD Leaders indicate that anxiety is a significant problem for some of the school-age children being provided services at ASD community-based agencies. Interviews with ASD Leaders regarding current use of CBT by ASD-CPs found only two of the five agencies using CBT strategies to treat anxiety among school-age children with ASD. Of these, one agency reported using a manualized anxiety CBT developed for typically developing children with anxiety disorders rather than developed for children with ASD, and the other used exposure therapy (a component of CBT) but indicated that ASD-CPs were not certain about how to effectively use this practice with children with ASD. Using the AMI to implement a specific EBP to treat anxiety disorders in school-age children with ASD, aim three will evaluate the: feasibility of using the AMI within ASD community-based agencies; perceived fit, relevance, utility and compatibility of the AMI within ASD community-based agencies; training and support in the use of the AMI and AMI process materials; and observed fidelity of using the AMI to implement at EBP within ASD community-based agencies.

### Participants

Approximately 10 ASD Leaders (*e.g.*, clinical directors, executive directors), not participating in the ACC, will be recruited to participate in the AMI pilot study to test the feasibility and acceptability of the AMI. An agency will be eligible if it provides behavioral interventions, speech and language therapy, or social skills training to children with ASD and co-occurring anxiety disorders between the ages of 7–11 years. Agencies will be excluded if they do not provide services to children aged 7–11 years or do not serve any children with co-occurring anxiety disorders.

### Procedure

The AMI will be used to implement ‘Building Confidence,’ a manualized CBT for children with ASD and co-occurring anxiety disorders [49]. The PI is a co-author of the manualized intervention and will consult with the primary author throughout the pilot study to address EBP factors (*e.g.*, time available for training, billing procedures, *et al*.), organizational adoption-related factors, and factors facilitating implementation. The pilot test will involve a pre-post design. ASD Leaders will be asked to complete questionnaires and participate in interviews prior to and after implementation of the AMI. Measures of AMI implementation will involve process and fidelity measures within each agency.

### Data/measures

Data from the demographic survey, EBPAS, CSS, and PSS used during aim one will be administered at pre- and post-implementation of the AMI (see aim one for more information about these measures).

### Stages of implementation completion (SIC)

The SIC [50] is a time-to-event based observational measure of implementation progress that consists of eight stages relating to specific implementation milestones. These milestones include: engagement, consideration of feasibility, readiness planning, staff hired/trained, adherence monitoring processes in place, services and consultation begin, ongoing services, consultation fidelity monitoring and feedback, and competency. The milestones span the timeframe from the engagement stage where initial contact between interested parties occurs through the attainment of program competency stage. Stages extend from ASD Leaders through ASD-CPs in order to capture the multiple levels involved in implementation. The SIC is intended to be tailored to fit specific EBPs and implementation models, and can be modified to assess quality and fidelity of the stages of implementing the AMI. The measure authors have agreed to work with the PI to adapt the SIC to best reflect the phases and steps within the AMI. Analysis of the SIC involves a time-to-event modeling method.

### Individual interviews

Leaders will be interviewed prior to the AMI implementation to assess their perceptions of the AMI, including perceived fit, relevance, utility, and compatibility with their ASD agency. At post-implementation, interviews will assess perspectives about the AMI, including feasibility and acceptability, changes in agency process for implementing EBPs, and changes in ASD-CP practices. Individual interviews will take approximately 30 minutes.

### Analysis plan: Integration of qualitative and quantitative analyses

Repeated measures ANOVA for pre- and post-implementation questionnaires will be used to examine the changes that occurred during AMI. Due to the lack of a control group and the small sample sizes, interpretation of effect sizes will be examined.

The SIC will be analyzed with guidance from the measure authors and statistical consultant using the Cox proportional hazards time-to-event modeling method [51] within multiple stages. Analyses will replicate those used during the measure development. In particular, an outside observer (*e.g.*, research assistant) will track the dates of completion of each activity within each phase of the AMI in a stage-tracking database. From these data, three scores are derived: the number of AMI phases completed, the time spent in each AMI phase, and the proportion of AMI Process Materials completed in each phase. For sites that choose to discontinue implementation, the discontinuation date will be logged accordingly in the furthest phase that the site entered. If this occurs, the time spent in the final phase of the AMI is calculated between the date of the earliest activity within the phase and the date of discontinuance. If the observation period ends before the phase is completed but a site has not discontinued implementation, the observation will be treated as being censored (*e.g.*, standard time-to-event analysis).

Pre- and post-implementation interviews will be analyzed using a coding, consensus, and comparison methodology [52], which follow an iterative approach rooted in grounded theory [53]. Transcriptions will be independently coded by the PI and a trained research assistant at a general level in order to condense data into analyzable units. The coding will be reviewed by grant mentors and consultants. Transcriptions will be assigned codes based on a priori or emergent themes. Transcriptions may be assigned more than one code. Disagreements will be resolved through subsequent discussion between research team members. The final list of codes will be developed through consensus with ACC partners. Based on the codes, the process of axial coding will be used to generate a series of categories arranged in a treelike structure connecting segments into separate categories or nodes. Nodes and trees will be used to create a taxonomy of themes, including *a priori* and emergent categories and new, unrecognized categories. Results will be presented to ACC partners for interpretation.

Finally, methods for integrating qualitative and quantitative data for analyses will be the same as those used in aim one (see aim one for details).

## Discussion

The AMI will represent a data-informed approach to facilitate implementation of EBPs by ASD-CPs by providing an implementation model specifically developed for this context. This study is designed to address the implications of EBP implementation in ASD community-based agencies. In doing so, the exploratory and developmental work proposed in this study aims have a positive public health impact on the lives of children with ASD and their families by increasing the implementation of EBPs by ASD-CPs, especially for children with ASD with co-occurring anxiety disorders. Moreover, the proposed study will positively impact the field of implementation science by providing an empirically-supported and tested model of implementation to facilitate the identification, adoption, and use of EBPs by ASD-CPs, specifically.

In this project, the AMI will be developed and pilot tested. The AIM is designed to assist ASD community-based agencies assess and find EBPs to their meet agency needs, systematically evaluate the EBP for acceptability and feasibility within the agency, consider salient adoption factors, and guide the effective implementation of the EBP. Through the use of the AMI phases and AMI process materials (*e.g.*, structured materials guiding the phases of implementation), it is hypothesized that implementing EBPs will be more likely for ASD community agencies.

Once developed and pilot tested, further refinement of the AMI, including specific aspects of the AMI process materials, will be done to allow for larger-scale implementation studies focusing on testing the implementation effectiveness, generalizability, and sustainability of the AMI. By continuing to involve ACC partners, the external validity of refinements made to the AMI will increase. Additionally, larger-scale studies will allow for involving more varied organizations or systems providing services to children with ASD (*e.g.*, school districts) as well as diverse providers, and multilevel data (*e.g.*, ASD-CPs nested within managers nested within agencies nested within service systems). Future tests of the effectiveness of the AMI will not have an EBP selected a priori.

## Abbreviations

ASD-CP: Autism spectrum disorders community provider; ASD: Autism spectrum disorder; AMI: Autism Model of Implementation; CBT: Cognitive-behavioral therapy; CSS: Children’s Services Survey; DTT: Discrete trial teaching; EBP: Evidence-based practice; EBPAS: Evidence-based Practice Attitude Scale; PI: Principal investigator; PSS; ASD Community Provider Strategies Survey; SIC; Stages of Implementation Completion.

## Competing interests

GAA is an Associate Editor of Implementation Science; all decisions on this paper were made by another editor. The authors declare that they have no other competing interests.

## Authors’ contributions

AD is the principal investigator for the described study. AD conceptualized and designed the study, drafted the manuscript, and approved the final version. GAA and AS are co-mentors for AD’s NIMH K01 award, which funds the study activities. GAA and AS contributed to the conceptualization and design of the study, reviewed and provided feedback on the manuscript. All authors read and approved the final manuscript.
